# The Role of Elevated Wall Shear Stress in Progression of Pulmonary Vein Stenosis: Evidence from Two Case Studies

**DOI:** 10.3390/children8090729

**Published:** 2021-08-25

**Authors:** Peter E. Hammer, Kerry McEnaney, Ryan Callahan, Christopher W. Baird, David M. Hoganson, Kathy J. Jenkins

**Affiliations:** 1 Department of Cardiac Surgery, Boston Children’s Hospital, Boston, MA 02115, USA; Chris.Baird@cardio.chboston.org (C.W.B.); David.Hoganson@cardio.chboston.org (D.M.H.); 2 Department of Cardiology, Boston Children’s Hospital, Boston, MA 02115, USA; kerry.mcenaney@childrens.harvard.edu (K.M.); Ryan.callahan@cardio.chboston.org (R.C.); kathy.jenkins@cardio.chboston.org (K.J.J.)

**Keywords:** pulmonary vein stenosis, wall shear stress, neointimal hyperplasia

## Abstract

Pulmonary vein stenosis is a serious condition characterized by restriction or blockage due to fibrotic tissue ingrowth that develops in the pulmonary veins of infants or children. It is often progressive and can lead to severe pulmonary hypertension and death. Efforts to halt or reverse disease progression include surgery and catheter-based balloon dilation and stent implantation. Its cause and mechanism of progression are unknown. In this pilot study, we propose and explore the hypothesis that elevated wall shear stress at discrete pulmonary venous sites triggers stenosis. To assess this theory, we retrospectively analyzed cardiac catheterization, lung scan, and X-ray computed tomography data to estimate wall shear stress in the pulmonary veins at multiple time points during disease progression in two patients. Results are consistent with the existence of a level of elevated wall shear stress above which the disease is progressive and below which progression is halted. The analysis also suggests the possibility of predicting the target lumen size necessary in a given vein to reduce wall shear stress to normal levels and remove the trigger for stenosis progression.

## 1. Introduction

Pulmonary vein stenosis (PVS) is a critical narrowing of one or more pulmonary veins that affects 1.7 per 100,000 children under the age of two [1]. PVS can arise following surgical correction of abnormal pulmonary venous drainage in infants, or it can arise without apparent cause as a primary condition in normally connected vessels. Stenosis often originates where a pulmonary vein discharges into the left atrium. Other origins include a surgical connection or sites where an external structure such as an airway impinges on a pulmonary vein. Initial stenosis typically takes the form of neointimal hyperplasia that develops into an annular restriction [2,3]. Stenosis can progress to upstream segments of that branch, manifesting as a diffuse and gradual decrease of vessel caliber. The disease can further progress to involve other branches of the same pulmonary vein, or to other pulmonary veins, resulting in pulmonary hypertension, heart failure, and death. A hallmark of the disease is recurrence or progression, even after relief of stenosis with surgery or catheter-based intervention. No treatments, including medical, surgical, or catheter based, are reliably effective in halting disease recurrence or progression. Limited understanding of the disease process and its underlying cause hinders the development of effective treatments.

Published reports have suggested that blood flow in the pulmonary veins is abnormal in patients who develop PVS. A meta-analysis of infants with primary PVS reported that the majority of infants with PVS had left-to-right shunts [4], indicating elevated flow through the pulmonary veins, and higher shunt exposure time is associated with the development of PVS in infants with Down’s syndrome [5]. High flow may also be associated with anemia or infection, and PVS is known to be associated with necrotizing enterocolitis [6] and other conditions related to prematurity [7,8]. Abnormal pulmonary venous flow can also arise at discrete sites where a vein is compressed, folded, or otherwise partially obstructed, and cardiologists and surgeons at our institution have observed that the initial site of stenosis is often associated with these areas of disturbed blood flow. Vein cross-sectional area is decreased at these sites producing a local increase in flow velocity. Increased overall pulmonary blood flow and the high-velocity flow through a deformed venous cross-section can, either individually or in combination, expose the vascular endothelial cells to a drag force, referred to as wall shear stress (WSS), that is abnormally high. 

While abnormally low levels of WSS have been strongly associated with pathological processes such as arterial plaque formation and in-stent restenosis [9,10,11], less is known about the effects of abnormally high WSS. However, published reports have shown that abnormally high levels of WSS trigger neointimal hyperplasia and progressive vascular stenosis. Binns et al. implanted PTFE grafts of different sizes in dog arteries and showed that the smallest grafts, which were exposed to a pathologically high WSS of 41 dyn/cm^2^, developed pseudointimal hyperplasia and loss of patency by 15 weeks [12]. Abnormally high WSS levels have also been associated with sites of artery stenosis caused by neointimal hyperplasia immediately distal to coronary stents [13]. Similar effects have been observed in veins. For example, the arteriovenous grafts commonly implanted in dialysis patients often fail due to stenosis at the venous anastomosis resulting from neointimal hyperplasia. Fitts et al. reported neointimal hyperplasia at this site in response to high levels of WSS (>50 dyn/cm^2^) in a porcine model of arteriovenous grafts [14]. In a different study, Misra et al. calculated the average WSS at the stenotic region of the venous anastomosis to be 26 dyn/cm^2^, a value approximately 4 times that of the control vein [15]. While the precise level of WSS that triggers neointimal hyperplasia is unknown, the upper limit of venous WSS in iliac veins of normal, juvenile pigs has been reported to be approximately 10 dyn/cm^2^ [16], and this is in close agreement with our upper limits of WSS from unpublished estimates in normal pulmonary veins in children.

In this pilot study, we propose that abnormally high WSS in the pulmonary veins drives the occurrence and progression of PVS, and we assess this hypothesis by retrospectively analyzing serial cardiac catheterization and computed tomography (CT) data in two patients that suffered long courses of the disease. We use these data to calculate WSS in each pulmonary vein at its left atrial confluence at multiple time points during the treatment of the disease. We then interpreted the effects of interventional treatments on disease progression in light of these periodic WSS estimates. We also propose a simple, quantitative approach to predicting target vessel lumen dimensions to reduce WSS to normal levels and prevent restenosis. 

## 2. Materials and Methods

### 2.1. Calculation of Wall Shear Stress from Clinical Data

For steady, laminar blood flow in large, cylindrical vessels, wall shear stress, *τ*, can be calculated as follows:

(1) $$\tau =\frac{4\mu \bar{v}}{r}$$

where *µ* is the viscosity of blood, *r* is vessel radius, and *v* is the spatial average of blood flow velocity across the vessel cross section [17]. Average velocity can be calculated as volumetric flow rate through the vessel divided by vessel cross-sectional area. For vessels of elliptical cross section, this is calculated as follows:

(2) v¯=Qπab

where *a* and *b* correspond to half of the major and minor axes of the ellipse, respectively. Wall shear stress will vary around the perimeter of an elliptical vessel with its minimum corresponding to the major axis and its maximum corresponding to the minor axis. It can be calculated by substituting *a* or *b* for r in Equation (1). Using these relationships, the diameter of the circular vessel required to achieve a critical value of WSS for a given flow rate and viscosity is

(3) $$d_{crit}=2\sqrt[{3}]{\frac{4\mu Q}{\pi \tau_{crit}}}$$

These expressions for WSS are only valid if blood flow in the vessel is laminar flow, and this condition holds when Reynold’s number is less than approximately 2000 [15]. Reynold’s number is a dimensionless quantity that represents the ratio of inertial to viscous forces in moving fluid and can be calculated as follows:

(4) $$Re=\frac{\bar{v}d_{H}\rho }{\mu }$$

where *d_H_* is the hydraulic diameter of the vessel (i.e., vessel cross-sectional area divided by perimeter), and *ρ* is the density of blood.

### 2.2. Case 1

Patient 1 (Pt1) was born at full term and was diagnosed at 1 month of age with pulmonary hypertension. The patient was referred to our institution at 2.5 months of age with pulmonary hypertension and severe pulmonary vein stenosis. The patient was known to have a large secundum ASD, bilateral SVCs with a persistent left SVC to an intact coronary sinus with no bridging vein. Pulmonary vein disease was assessed with catheterization, demonstrating no more than mild stenosis of the veins going to the right and left lower lobes, evidence of significant obstruction of the left upper lobe pulmonary vein, and almost complete occlusion of the right upper pulmonary vein. The patient underwent surgery at 3 months of age that involved resection of pulmonary vein ostial stenoses in right lower, left upper, and left lower pulmonary veins, and the left atrium was reconstructed with a pericardial well (sutureless type pulmonary vein repair). The ASD was repaired with autologous pericardium with a 4 mm fenestration.

Over the subsequent 2 years, the patient underwent 12 cardiac catheterizations to balloon dilate and/or stent stenotic segments and had 5 CT scans to assess the pulmonary veins. The patient also received targeted antiproliferative drug therapy with imatinib and bevacizumab as well as two 8-week courses of sirolimus for in-stent restenosis. The disease progressed despite treatments, and the patient died at age 2 years and 8 months due to complications of severe pulmonary hypertension. 

The dimensions of the three patent pulmonary veins at their confluences with the left atrium were measured from CT at the time of each scan, and the cardiac index, body surface area, and Q_P_:Q_S_ ratio were measured by cardiac catheterization within a month prior to each CT. The distribution of pulmonary blood flow to left and right lungs was measured by lung scan. The total blood flow entering the left atrium through the single right pulmonary vein ostium was calculated as the product of the cardiac index, body surface area, and Q_P_:Q_S_ ratio times the proportion of pulmonary flow to the right lung. The blood flow through each of the two left pulmonary veins was calculated in a similar way, with the total flow to the left lung divided equally between the left upper and left lower pulmonary veins.

### 2.3. Case 2

Patient 2 (Pt2) was born at 35 weeks and was noted to have an 8 mm secundum ASD, a persistent left SVC to an intact coronary sinus (no bridging vein), and a small muscular VSD that later closed spontaneously. At 4 months of age, the patient presented in respiratory distress and was noted to have a dilated RV and right upper lobe atelectasis. Subsequent cardiac catheterization demonstrated RV hypertension, absence of the right upper and right middle lobes of the lung, and left upper and right pulmonary vein stenosis, both of which were balloon dilated.

On cardiac catheterization at 6 months, the left upper and right pulmonary veins were again found to be stenotic, and the patient underwent surgery for PVS repair. The left upper and right pulmonary veins were opened out to the entry point into the pericardium, and transverse cuts on the left atrium were sewn to the pericardium to create a sutureless repair. The ASD was repaired with autologous pericardium, leaving a 3 mm fenestration.

Following surgery, the disease persisted for approximately 7 years during which the patient underwent 11 cardiac catheterizations for balloon dilation (BD) and/or stent placement in stenotic segments and underwent 8 CT scans. The patient also received imatinib therapy and one 8-week course of sirolimus. Following the last catheter intervention, the PVS had resolved, and no evidence of stenosis was observed at the most recent follow-up CT. Pulmonary vein dimensions and blood flow were calculated at the time of CT scans based on CT and catheterization data, as were performed for Pt1. 

## 3. Results

### 3.1. Case 1

For Pt1 at the time of each CT scan, the major and minor diameters of the three patent pulmonary veins at their confluences with the left atrium appear in Table 1, along with data from cardiac catheterization including the patient’s body surface area and Q_P_:Q_S_ ratio and pulmonary blood flow distribution to left and right lungs from a lung scan. Calculated values of WSS and Reynold’s number are listed, as is the vessel diameter required to produce a WSS of 10 dyn/cm^2^—chosen as the upper limit of normal. In all cases, Reynold’s number was well below 2000, indicating an absence of true turbulent flow.

**Figure 1:**
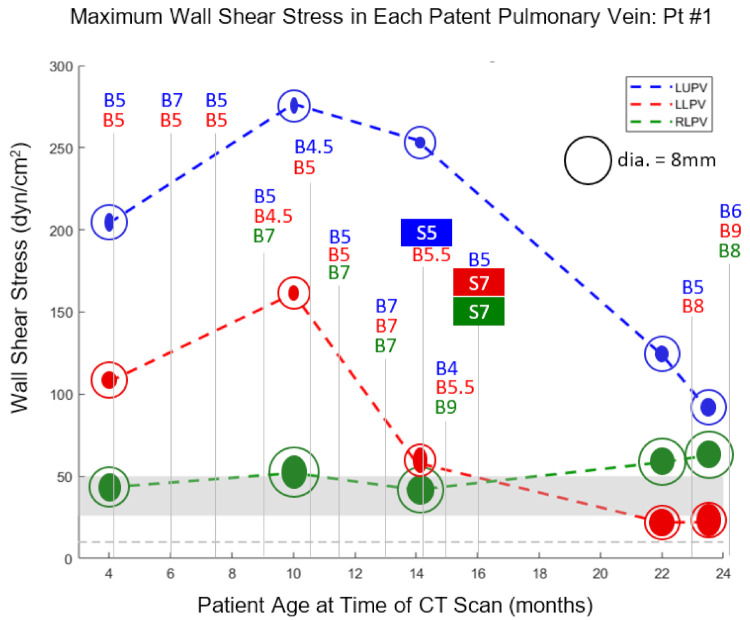
For Pt1, maximum WSS in the LUPV, LLPV, and RLPV is plotted versus patient age at the time of each CT scan. The marker for each plotted point consists of a solid ellipse, whose major and minor diameters correspond to those of the vein as measured by CT, along with a circle with diameter corresponding to the target lumen size required to achieve a WSS of 10 dyn/cm^2^. Each balloon dilation (B) is labeled at the appropriate point on the timeline along with the maximum balloon diameter (mm) that was achieved. Stent (S) implantation is similarly labeled along with stent diameter in mm. The gray horizontal bar indicates the WSS range between 26 and 50 dyn/cm^2^ at which neointimal hyperplasia has been reported to develop in large veins. The gray horizontal dotted line indicates a WSS level of 10 dyn/cm^2^, taken as the upper limit of normal.

WSS values were plotted as functions of time (patient age) along with times and maximum diameters of catheter-based balloon dilations and vessel stenting (Figure 1). It is important to note that the diameter of the largest balloon inflated in a stenotic PV is not necessarily the size of the patent lumen after the balloon is deflated and withdrawn. On occasions when lumen size was measured angiographically immediately following balloon deflation, it was consistently between 20 and 50% smaller than the maximum size of the inflated balloon, indicating substantial vessel recoil. 

The first CT scan for Pt1 (4 m) was approximately 5 weeks after the sutureless repair, and WSS levels can be seen to be severely elevated in the left upper pulmonary vein (LUPV) and left lower pulmonary vein (LLPV) and somewhat elevated in the right lower pulmonary vein (RLPV). During the subsequent six months, the LUPV underwent BD four times, to either 5 or 7 mm, and the LLPV also underwent BD four times, to 5 mm. Despite these interventions, WSS in all three patent PVs was further elevated by the second CT scan, and the absolute size of both the LUPV and LLPV decreased during this time, concurrent with flow redistribution away from the left lung (Table 1), even though BSA increased by 36%. A large discrepancy can be noted between the PV dimensions measured from CT and the diameter required to keep WSS at or below 10 dyn/cm^2^ (Figure 1). 

Three more BDs over the next four months decreased WSS in all three PVs, although they all remained elevated. Stenting of the LUPV immediately following the 14 m CT led to a substantial drop in WSS, though it remained severely elevated at over 100 dyn/cm^2^ based on the final 2 CT scans. Aggressive stenting and BD of the LLPV following the third CT scan was effective in reducing WSS in that vessel to 22 dyn/cm^2^, and measurement of the LLPV at the last two CT scans indicates that another 1–2 mm of vessel dilation would have restored WSS to 10 dyn/cm^2^ or less (Table 1). The RLPV, despite stenting (7 mm) and BD (8 mm), continued to exhibit a minor diameter of approximately 4.5 mm, considerably less than the 8 mm diameter predicted to produce a normal level of WSS. Although WSS estimates were trending downward in two of the three PVs over the course of the last few CT scans, they were still severely elevated in two of the three PVs, and the patient’s condition declined. 

### 3.2. Case 2

**Figure 2:**
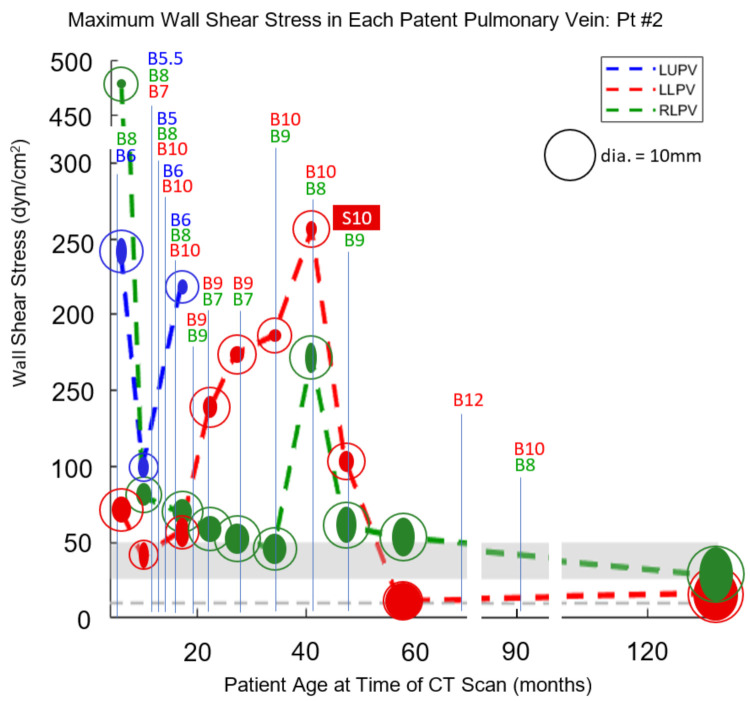
For Pt2, maximum WSS in the LUPV, LLPV, and RLPV is plotted versus patient age at the time of each CT scan. The marker for each plotted point consists of a solid ellipse, whose major and minor diameters correspond to those of the vein as measured by CT, along with a circle whose diameter corresponds to the target lumen size required to achieve a WSS of 10 dyn/cm^2^. Each balloon dilation (B) is labeled at the appropriate point on the timeline along with the maximum balloon diameter (mm) that was achieved. Stent (S) implantation is similarly labeled along with stent diameter in mm. The gray horizontal bar indicates the WSS range between 26 and 50 dyn/cm^2^ at which neointimal hyperplasia has been reported to develop in large veins. The gray horizontal dotted line indicates a WSS level of 10 dyn/cm^2^, taken as the upper limit of normal.

For Pt2, pulmonary vein dimensions and catheterization data appear in Table 2, along with calculated values of WSS, Reynold’s number, and the vessel diameter required to produce a WSS of 10 dyn/cm^2^. Reynold’s number remained below 2000 in all cases, as it did for Pt1, indicating that the laminar flow conditions assumed for Equations (1) and (3) are valid. Wall shear stress values were plotted as functions of time (patient age) along with times and maximum diameters of catheter-based balloon dilations and vessel stenting (Figure 2).

The first CT scan for Pt2 was one day prior to sutureless repair, and a large drop in WSS levels can be seen at the time of the second CT scan three months later (Figure 2), although levels are still elevated in all three vessels. By the third CT scan seven months later, Pt2 had undergone three more catheterizations for worsening symptoms, and despite aggressive BD, WSS remained elevated in all three vessels. In particular, WSS underwent a large rise in the RLPV, and by the subsequent catheterization 2 months later, the RLPV was atretic. This loss of patency would require all pulmonary blood to flow through the left-sided PVs, and indeed the LLPV experienced a large jump in WSS during this interval. Over the next 18 months, frequent BD was used to combat persistent severe stenosis in the LLPV, which finally appeared to resolve when a 10 mm stent was implanted at 47 m and dropped WSS to near-normal levels. BD of this LLPV and the somewhat less stenotic LUPV over the next 5 years appeared to result in near normalization of WSS levels and resolution of the disease.

## 4. Discussion

This analysis of blood flow through pulmonary veins shows that WSS levels are elevated in vessels with known PVS and are in a range that has been shown to trigger neointimal hyperplasia in large veins. While this study does not irrefutably demonstrate that elevated WSS causes the development and progression of PVS, it does support elevated WSS as a plausible explanation that can account for several observations of the disease. 

**Figure 3:**
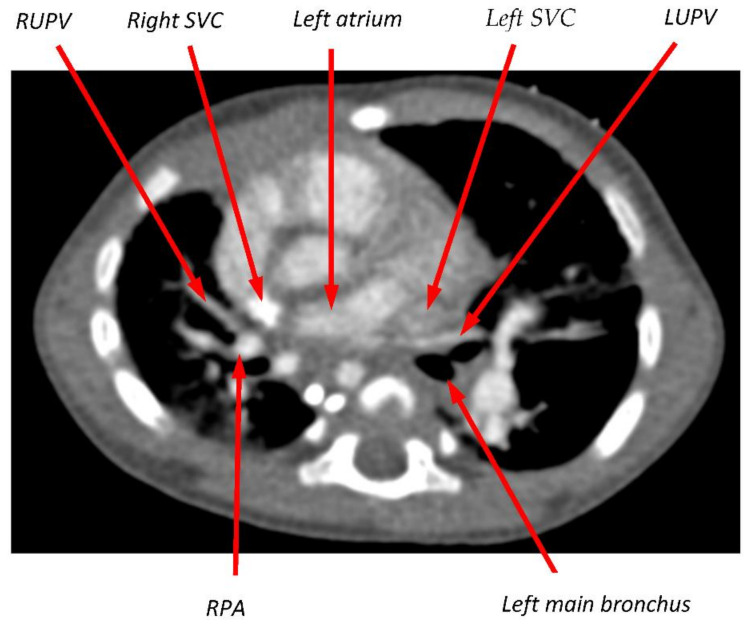
Transverse slice of CT scan from Pt2 at the level of the left atrium (LA) and upper pulmonary veins. The RUPV was documented as atretic at the time of this scan, but its course had presumably been between the right superior vena cava (SVC) and the right pulmonary artery (RPA). On the left side, the takeoff of the LUPV from the LA is sharply angulated, and the vein appears compressed as it passes between the left SVC and the left main bronchus.

In the case of Pt2, the stenting of the LLPV to 10 mm and follow-up BDs achieved durable lumen dimensions of 10.0 × 8.6 mm, quite close to the value of 10.8 mm predicted by this theory to result in normal WSS. Our theory also ties together two phenomena that are associated with PVS—elevated pulmonary blood flow and a compressed or partially obstructed pulmonary vein. Both directly increase WSS. In both Pt1 and Pt2, PVS and pulmonary hypertension were present, and the right upper pulmonary veins were atretic by the first CT scan, making it impossible to assess the role of elevated WSS in the early development of disease. However, the 6-month CT scan from Pt2, acquired just prior to sutureless repair, shows clear evidence of compression of the RUPV between the right SVC and the right pulmonary artery and for compression of the LUPV between the left SVC and the left main bronchus (Figure 3). This lumen compression, combined with elevated pulmonary blood flow due to the large ASD, makes it likely that WSS was elevated in the RUPV, possibly triggering stenosis that progressed ultimately to atresia. Vein atresia leads to flow redistribution to less affected pulmonary veins, increasing flow and potentially stimulating further disease. The hypothesized causative role of distorted proximal pulmonary veins and elevated pulmonary blood flow is consistent with our prior finding that progressive PVS after cardiac surgery is more strongly associated with intrinsic factors, such as atrial and pulmonary venous anatomy, than with an external insult at the time of surgery [18]. 

**Figure 4:**
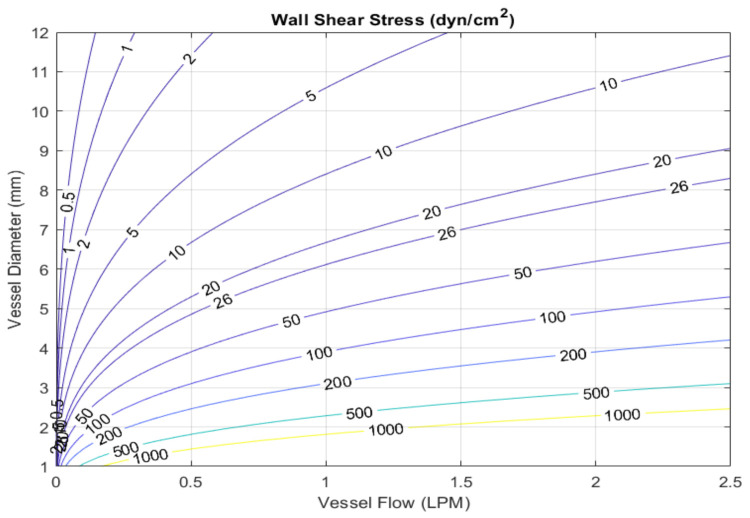
Contour plot showing the relationship between blood flow rate through a vessel, the vessel diameter, and wall shear stress. For example, in a pulmonary vein with 1 LPM of flow, in order to keep WSS below 10 dyn/cm^2^ the vessel diameter must be at least 8.4 mm.

Our analysis suggests a quantitative method based on WSS for treating PVS. By achieving durable dilation of a stenotic vessel to an adequate lumen size, the stimulus for neointimal hyperplasia and further stenosis may be eliminated. In fact, if a target level of normal WSS is known, then it is possible to calculate the target size for BD or stenting given the amount of flow that the vein must carry (Figure 4). For example, if the total pulmonary blood flow of 2.5 LPM in a given patient is divided equally among five pulmonary veins, then the diameter of those vein confluences must be approximately 7 mm or greater to keep WSS below 10 dyn/cm^2^ (Figure 4). Furthermore, in addition to using the pressure gradient measured by echocardiography to assess the efficacy of BD, fluoroscopic measurement of the post-PD lumen size can be used to determine whether WSS has been restored to normal levels. Persistently elevated WSS following surgical or transcatheter intervention may lead to decisions in clinical management, including repeat interventions or possible use of targeted antiproliferative therapy [19,20]. 

The analysis method used in this study is relatively simple and is based on assumptions that might not strictly hold in some cases. For example, flow into the left atrium is not purely steady, and neglecting cyclic variation in the flow velocity in a vein will likely underestimate maximal flow velocity and consequently maximal WSS. Additionally, the WSS calculation is based on axisymmetric flow, and this too might not strictly apply—for example, for PVs that curve as they enter the left atrium. Here, too, it is likely that our calculated peak WSS values lead to an underestimate. 

Our method for estimating WSS depends not only on adequate imaging quality but also on the accuracy of catheterization data and related assumptions. For example, the WSS in a vein is proportional to flow, which is calculated based on cardiac index, Q_P_:Q_S_, and lung scan results, all of which have accuracy limitations. Furthermore, while lung scan data were used to provide an approximate value for the flow split to right and left lungs, the flow split among the lobes of a given lung was assumed to be equally divided for the purposes of this study. Fortunately, errors in the flow-based variables have a much smaller effect on WSS than errors in vessel dimensions, which influence WSS with the inverse third power of vessel dimensions. Another important study limitation is that we do not have data on the range of WSS in normal pulmonary veins in infants and children, although such data would not be difficult to collect. We also do not know a precise threshold value of WSS above which neointimal hyperplasia and stenosis have been demonstrated to develop. In fact, in the absence of a proposed mechanism linking elevated WSS to neointimal hyperplasia, it is difficult to know for certain that an explicit threshold exists. 

In conclusion, the theory that elevated WSS drives the progression of PVS is consistent with the details of disease progression in two clinical cases of severe, progressive PVS in this pilot study. The quantitative approach based on WSS levels that we propose as a guide for dilating and/or stenting veins to halt the progression of stenosis is both simple and testable, and efforts will continue to assess the validity of the proposed relationship between WSS and PVS development and progression. 

## Figures and Tables

**Table 1:** Data from Pt1 used to compute wall shear stress at the confluences of the pulmonary veins with the left atrium. Columns, from left to right, are age in months at time of CT scan, body surface area (BSA) in m^2^, cardiac index (CI) in L/min/m^2^, pulmonary to systemic blood flow ratio (Q_P_:Q_S_), left to right lung blood flow ratio, dimensions of pulmonary vein confluence at narrowest point (major diameter, 2a, and minor diameter 2b, in mm), maximum wall shear stress, corresponding to minor diameter b (τ), Reynold’s number (dimensionless), and estimated diameter of pulmonary vein segment necessary to reduce wall shear stress to 10 dyn/cm^2^ (d_tau = 10_).

Age Months	BSA m^2^	CI L/min/m^2^	Q_p_:Q_s_	Q_L_/Q_R_	2a mm	2b mm	Τ dyn/cm^2^	R_e_	d_τ__= 10_ mm	
4	0.22	3.2	1.8	58/42	3.3	1.8	204	898	6.0	LUPV
					3.0	2.6	108	835	6.0	LLPV
					4.8	3.9	43	777	6.8	RLPV
10	0.30	2.8	1.8	35/65	2.9	1.4	277	760	5.4	LUPV
					2.7	1.9	161	727	5.4	LLPV
					5.8	4.4	52	1221	8.4	RLPV
14	0.33	4.0	1.0	40/60	1.9	1.8	255	908	5.4	LUPV
					4.3	2.5	58	486	5.4	LLPV
					5.1	4.7	42	1029	7.8	RLPV
22	0.45	3.4	1.0	44/56	2.8	2.4	124	823	5.8	LUPV
					4.7	4.4	22	471	5.8	LLPV
					4.7	4.3	59	1212	8.0	RLPV
23.5	0.46	3.4	1.0	44/56	3.1	2.7	90	754	5.9	LUPV
					5.5	4.1	22	454	5.9	LLPV
					4.7	4.2	63	1252	8.0	RLPV

**Table 2:** Data from Pt2 used to compute wall shear stress at the confluences of the pulmonary veins with the left atrium. Columns, from left to right, are age in months at time of CT scan, body surface area (BSA) in m^2^, cardiac index (CI) in L/min/m^2^, pulmonary to systemic blood flow ratio (Q_P_:Q_S_), left to right lung blood flow ratio, dimensions of pulmonary vein confluence at its narrowest point (major diameter, 2a, and minor diameter 2b, in mm), maximum wall shear stress, corresponding to minor diameter b (τ), Reynold’s number (dimensionless), and estimated diameter of pulmonary vein segment necessary to reduce wall shear stress to 10 dyn/cm^2^ (d_τ = 10_).

Age Months	BSA m^2^	CI L/min/m^2^	Q_p_:Q_s_	Q_L_/Q_R_	2a mm	2b mm	τ dyn/cm^2^	R_e_	d_τ=10_ mm	
6	0.28	3.2	2.6	80/20	5.2	2.1	241	1554	8.2	LUPV
					5.1	3.9	71	1312	8.2	LLPV
					1.8	1.8	475	1648	6.5	RLPV
10	0.33	3.3	1.0	52/48	4.2	2.0	100	564	5.5	LUPV
					5.2	2.0	40	435	5.5	LLPV
					4.3	3.0	80	905	6.8	RLPV
17	0.39	4.2	1.0	52/48	2.9	2.0	218	1097	6.3	LUPV
					5.6	2.8	58	628	6.3	LLPV
					4.9	3.7	70	1158	7.8	RLPV
22	0.42	5.2	0.8	44/56	-	-	-	-	-	LUPV
					4.3	2.8	135	1363	7.7	LLPV
					5.0	4.5	57	1310	8.3	RLPV
27	0.45	4.1	1.0	37/63	-	-	-	-	-	LUPV
					3.2	2.7	174	1471	7.4	LLPV
					5.8	4.7	54	1406	8.8	RLPV
					-	-	-	-	-	LUPV
34	0.48	3.0	1.0	34/66	2.5	2.5	186	1247	6.6	LLPV
					5.9	4.6	45	1148	8.3	RLPV
					-	-	-	-	-	LUPV
41	0.52	2.9	1.0	39/61	3.1	2.1	256	1427	7.0	LLPV
					6.0	2.3	172	1344	8.2	RLPV
					-	-	-	-	-	LUPV
47	0.55	3.1	1.0	35/65	4.0	2.9	105	1094	7.1	LLPV
					7.1	3.9	61	1256	8.7	RLPV
					-	-	-	-	-	LUPV
57	0.64	3.0	1.0	35/65	7.2	7.0	11	603	7.4	LLPV
					7.5	4.3	53	1322	9.1	RLPV
					-	-	-	-	-	LUPV
132	1.36	3.1	1.0	49/51	10.0	8.6	17	1412	10.7	LLPV
					10.4	6.6	28	1591	10.9	RLPV

## Data Availability

Not Applicable.
